# Walking Time Is Associated With Hippocampal Volume in Overweight and Obese Office Workers

**DOI:** 10.3389/fnhum.2020.00307

**Published:** 2020-08-20

**Authors:** Frida Bergman, Tove Matsson-Frost, Lars Jonasson, Elin Chorell, Ann Sörlin, Patrik Wennberg, Fredrik Öhberg, Mats Ryberg, James A. Levine, Tommy Olsson, Carl-Johan Boraxbekk

**Affiliations:** ^1^Department of Public Health and Clinical Medicine, Umeå University, Umeå, Sweden; ^2^Department of Integrative Medical Biology, Umeå University, Umeå, Sweden; ^3^Umeå Center for Functional Brain Imaging (UFBI), Umeå University, Umeå, Sweden; ^4^Department of Community Medicine and Rehabilitation, Umeå University, Umeå, Sweden; ^5^Department of Radiation Sciences, Umeå University, Umeå, Sweden; ^6^Mayo Clinic, Rochester, MN, United States; ^7^Fondation IPSEN, Paris, France; ^8^Danish Research Center for Magnetic Resonance (DRCMR), Centre for Functional and Diagnostic Imaging and Research, Copenhagen University Hospital, Hvidovre, Denmark; ^9^Institute of Sports Medicine Copenhagen (ISMC), Copenhagen University Hospital, Copenhagen, Denmark

**Keywords:** cognition, brain function, physical activity, sedentary behavior, office work, randomized controlled trial

## Abstract

**Objectives**: To investigate the long-term effects on cognition and brain function after installing treadmill workstations in offices for 13 months.

**Methods**: Eighty healthy overweight or obese office workers aged 40–67 years were individually randomized to an intervention group, receiving a treadmill workstation and encouraging emails, or to a control group, continuing to work as usual. Effects on cognitive function, hippocampal volume, prefrontal cortex (PFC) thickness, and circulating brain-derived neurotrophic factor (BDNF) were analyzed. Further, mediation analyses between changes in walking time and light-intensity physical activity (LPA) on changes in BDNF and hippocampal volume between baseline and 13 months, and multivariate analyses of the baseline data with percentage sitting time as the response variable, were performed.

**Results**: No group by time interactions were observed for any of the outcomes. In the mediation analyses, positive associations between changes in walking time and LPA on changes in hippocampal volume were observed, although not mediated by changes in BDNF levels. In the multivariate analyses, a negative association between percentage sitting time and hippocampal volume was observed, however only among those older than 51 years of age.

**Conclusion**: Although no group by time interactions were observed, our analyses suggest that increased walking and LPA may have positive effects on hippocampal volume and that sedentary behavior is associated with brain structures of importance for memory functions.

**Trial Registration**: www.ClinicalTrials.gov as NCT01997970.

## Introduction

During the last decades, an increase in sedentary behavior has occurred, particularly within the work domain where office workers are often engaged in excessive sedentary behavior. Sedentary behavior is defined as “all waking activities performed in a sitting, lying or reclining posture, with an energy expenditure of 1.5 Metabolic Equivalents (METs) or less” (Tremblay et al., 2017). The associated decrease of occupational-related energy expenditure could be one of the main reasons for the increase in body weight observed in the population during the last decades (Church et al., 2011). Sedentary behavior and physical inactivity are related to being overweight and obese, which in turn is related to brain health and cognitive functions. For example, Dahl and Hassing (2013) suggested that obesity in mid-life increases the risk of decreased cognitive function later in life. Also, obese individuals may perform less well concerning executive functions, working and episodic memory compared to normal-weight individuals (Loprinzi and Frith, 2018; Yang et al., 2018). Furthermore, a high body mass index (BMI) may be related with a greater decrease in brain volume (Brooks et al., 2013; Bobb et al., 2014; Walhovd et al., 2014), and a negative association between hippocampal volume and adiposity has been observed (Willette and Kapogiannis, 2015). Thus, there may be a link between obesity and certain brain regions including the prefrontal cortex (PFC) and the hippocampus, which in turn has a strong connection with different cognitive functions such as working memory, executive function, and episodic memory. However, these results mainly rely on observational data from cross-sectional studies.

The positive effects of aerobic exercise and cardiorespiratory fitness on cognitive function and brain structure are relatively well-studied (Colcombe et al., 2006; Erickson et al., 2011; Jonasson et al., 2017; Northey et al., 2018). However, less is known about the relationship between sedentary behavior, light-intensity physical activity (LPA), cognitive functions, and brain structure. Burzynska et al. (2014) showed that different levels of physical activity may have different associations with brain health. Specifically, it was observed that the association between physical activity and white matter microstructural integrity varied across different regions of the brain. Further, a systematic review of observational studies (Falck et al., 2017) suggested that excessive sedentary behavior is associated with lower cognitive performance, such as memory, executive function, and global cognition. A recent study, based on analyses of five different cohorts with one using sensor-based measurements of sedentary behavior, observed no overall support for associations between sedentary behavior and cognition, although it was implied that certain types of sedentary behavior may be differentially associated with cognitive performance (Maasakkers et al., 2020). This is further supported in other studies, showing that while TV viewing time has been associated with poorer cognitive function, the internet, and computer use has been associated with better cognitive functions (Kesse-Guyot et al., 2012; Hamer and Stamatakis, 2014). The relationship between sedentary behavior and the brain is thus complex, probably relying on multiple factors including physical activity, anthropometric and metabolic factors, and needs to be further investigated. One potential mediating factor between exercise and neural plasticity is brain-derived neurotrophic factor (BDNF; Erickson et al., 2011), but putatively mediating factors between sedentary behavior, lower intensities of physical activity and neural plasticity have not been established. Further, most studies investigating associations between sedentary behavior and cognition have used subjective measures of sedentary behavior, with questionable validity and reliability. Studies using sensor-based measurements of sedentary behavior and LPA are thus warranted.

Decreasing sedentary behavior in exchange for LPA could be one way to improve cognitive health. Interestingly, it has been observed that one 10-min single bout of very light-intensity exercise in healthy young adults can enhance memory function and functional connectivity of the hippocampus and cortex (Suwabe et al., 2018), although this has only been studied in a laboratory setting. However, another non-randomized study observed no effects on cognitive function from installing bike desks at offices for 5 months (Torbeyns et al., 2016). Likewise, while exchanging sedentary time for moderate- to vigorous physical activity (MVPA) improved executive function in healthy older adults, exchanging sedentary time to LPA did not show any significant effects (Fanning et al., 2017). This emphasizes the need for randomized controlled trials (RCT) with long-term follow-ups that would study the effects of changing occupational patterns of sedentary behavior and LPA on cognition and brain volume.

One approach to increasing physical activity in office environments is to install treadmill workstations, whereby office workers can walk while doing work at their desks. To date, no RCT exists examining the effects of treadmill workstations on cognitive function and brain structure. We have previously shown that, by installing treadmill workstations, sensor-measured daily time spent walking is increased over 13 months in overweight and obese office workers (Bergman et al., 2018). In the present sub-study, we examined whether the increased time spent walking influenced cognitive performance, hippocampal volume, and PFC thickness. Our theoretical point of departure stems from the underlying process logic (Greenwood and Parasuraman, 2016), which posits that observable changes in brain structure are more likely to influence the cognitive functions relying upon processing in those same structures. Our cognitive test battery emphasized episodic memory, executive functioning, and working memory. We, therefore, decided to focus the analyses on the hippocampus and three frontal regions, i.e., dorsolateral PFC (dLPFC), ventrolateral PFC (vLPFC), and anterior cingulate cortex (ACC). Based on evidence from both experimental and observational research presented above, showing positive effects of aerobic exercise on brain structure (Colcombe et al., 2006; Erickson et al., 2011; Jonasson et al., 2017; Falck et al., 2017; Northey et al., 2018), we hypothesized that the installation of the treadmill workstations, with increased time spent walking and LPA, would lead to improved cognitive performance and an increase in hippocampal volume and PFC thickness compared to the control group. We also hypothesized that putative changes in hippocampal volume observed by increasing time spent walking would be mediated by increased BDNF levels due to its involvement as a mediating factor between exercise and neural plasticity (Erickson et al., 2011). Finally, as the relationship between sedentary behavior and the brain is complex, probably relying on multiple factors, we wanted to explore associations between sedentary behavior, physical activity, and different body and brain measurements, using orthogonal partial least squares (OPLS) analyses on the baseline data.

## Materials and Methods

### Study Design

The present study is a part of the Inphact Treadmill study, described in detail elsewhere (Bergman et al., 2015, 2018). Briefly, the study was an RCT including healthy office workers between 40–67 years of age, having mainly sedentary work tasks, an adjustable sit-stand office desk available at their workspace, and a BMI between 25 and 40 kg/m^2^. All included participants were individually randomized to either an intervention (*n* = 40) or a control group (*n* = 40). Nineteen participants from the intervention group and 21 participants from the control group were randomly assigned for magnetic resonance imaging (MRI) measurements. Statistical power was calculated for daily walking time, the primary outcome in the Inphact Treadmill study. The study was approved by the Regional Ethical Review Board, Umeå, Sweden, and all participants gave both oral and written informed consent to participate. The trial is registered at www.clinicaltrials.gov as NCT01997970.

After randomization, all participants received a standard health consultation regarding diet and physical activity from a trained nurse, together with feedback on some of their screening and baseline measurements. Participants in the control group continued to work as usual at their sit-stand office desk. Participants in the intervention group had a treadmill workstation installed at their sit-stand office desk, which they were asked to use at least 1 h per day during the entire intervention which lasted 13 months. On four occasions, encouraging e-mails were sent to the intervention group.

### Outcomes

Participants visited the University hospital of Umeå and Umeå University, Sweden, on two separate days for the cognitive tests and the anthropometric, body composition, and metabolic measurements, respectively, at baseline, after 6 months and 13 months. Participants randomized for the MRI measurements visited the University Hospital on a third-occasion at baseline and 13 months. Accelerometer data were collected at baseline and after 2, 6, 10, and 13 months.

#### Cognitive Tests

Cognitive function was measured using a condensed version of the cognitive tests described by Jonasson et al. (2017). Four trained psychology students performed cognitive tests during office hours at Umeå University, Sweden. The total time for the tests was about 45 min. No restrictions were used before the cognitive tests, but participants were asked to sleep and eat as usual on the day before testing. To test episodic memory functions, we used free (immediate) recall (Murdock, 1962), and word recognition for delayed retrieval (Nyberg et al., 1996). To test executive function, we used N-back as a test of updating (Kirchner, 1958), Flanker test as a test of inhibition (Eriksen and Eriksen, 1974), and Trail making test (TMT) 2 and 4 as a test of shifting (Delis et al., 2001). The Backward digit span task was used to test working memory (Wechsler, 2010). To test processing speed, similarities task, and the digit symbol task from WAIS-R (both pen- and paper and computerized versions) were used (Wechsler, 2010). The order of the tests was free recall; n-back; recognition (encoding part); flanker test; backward digit span; trail making 2 and 4; digit symbol (computerized version); word recognition; similarities; and digit symbol (pen and paper version). The tests are described in detail in the study protocol (Bergman et al., 2015) and supplementary information. All computerized tests were presented using the E-Prime 2.0 software (Psychology Software Tools, Pittsburgh, PA, USA). Each test in the cognitive test battery was z-transformed based on their mean value and standard deviation of the entire study population at baseline. Tests where a low score indicates a better result were then multiplied by −1. To calculate the different cognitive domains (executive control, episodic memory, working memory, and processing speed) all tests measuring the same cognitive domain were used as unit-weighted composites. A total cognitive score was then calculated by averaging all four cognitive domains.

#### Magnetic Resonance Imaging (MRI) and Brain Segmentation

Structural imaging was performed at the University Hospital of Umeå, Sweden on a 3T Discover MR750 General Electric (GE) scanner equipped with a 32-channel head coil. T1-weighted structural images were collected with a 3D fast spoiled gradient echo sequence (180 slices with a 1 mm thickness, TR 8.2 ms, TE 3.2 ms, flip angle 12°, the field of view 25 × 25 cm, acquisition matrix 256 × 256 reconstructed to 512 × 512). Before the MRI measurements, participants were asked to avoid nicotine and caffeine for 1 h before the measurement and to avoid high-intensity exercise and alcohol for 24 h.

Freesurfer (Fischl et al., 2002) version 6 longitudinal stream (Reuter et al., 2012) was used to segment the brain. The volume of the left and right hippocampus (mm^3^) was derived from the subcortical segmentation and was combined into one measure. The hippocampus segmentations were further independently quality checked by two members of the research team, with manual editing of segmentations that did not fit against the hippocampus. No manual editing was performed on any of the segmentations and no participant was excluded based on the quality check. From the cortical segmentations, PFC thickness (mm) was derived. Similar to Vijayakumar et al. (2014), a dLPFC region-of-interest (ROI) included superior and middle frontal gyri; a vLPFC ROI included opercular, orbital and triangular gyri; and an ACC ROI included anterior and middle-anterior part of the cingulate gyri and sulci. The Left and right hemispheres were averaged for all PFC thickness measurements.

#### Sedentary Behavior and Physical Activity

Details on the instructions for wear and data processing of the accelerometer data were published previously (Bergman et al., 2018). Briefly, to measure sedentary behavior and physical activity intensity levels, activPAL, and ActiGraph were used. During each of the measurement periods, participants were also asked to report their usual sleeping hours and any longer time-period when not wearing one or both of the devices.

The activPAL3 and activPAL3 micro (default settings; PAL Technologies Limited, Glasgow, UK) were used on the thigh for 24 h per day for seven consecutive days, measuring time spent sitting, standing, and walking as well as sedentary behavior patterns. After visual inspection of the data, a custom Excel macro was used (HSC PAL analysis software v.2.19s) for calculation of the outcome measures. A time filter for total time awake on weekdays and weekends was applied based on the participant’s self-reported sleeping hours. Reported non-wear periods of >20 min were removed from the analyses.

During the same period when they were wearing the activPAL device, the participants also wore the ActiGraph wGT3x -BT (ActiGraph, Pensacola, FL, USA) around the waist. This device was worn for all waking hours for 14 consecutive days, measuring time in LPA and MVPA. Non-wear periods were defined as 60 min of consecutive zero counts, no spike tolerance, and a small window length of 1 min. Using the Actilife software (v.6.13.3), LPA was defined as 201–2,689 counts per minute and MVPA as 2,690 counts per minute or more.

#### Body Measurements, Blood, and Saliva Samples

As previously reported (Bergman et al., 2015), anthropometric measurements, including weight, height, waist and hip circumference, sagittal abdominal diameter, blood pressure, and resting heart rate, were measured using standardized methods. Body composition was assessed using Dual X-ray absorptiometry (DXA), measuring total body mass, fat mass, lean mass, and android and gynoid fat mass. Measurements were made after an overnight fast. Fasting blood samples were drawn for analysis of lipids, HbA1c, CRP, liver enzymes (AST, ALT), glucose, insulin, and BDNF. For salivary cortisol levels, participants chewed on a synthetic swab (Salivette Cortisol, Sarstedt, Nümbrecht, Germany) for 1 min at 7.00 am, 11.00 am 4.00 pm and 11.00 pm. The participants did this at home and were told to keep the saliva samples in their refrigerator until leaving the samples at the University Hospital in Umeå the following day. Saliva cortisol levels at the different time points were analyzed, and the area under the curve and the slope was calculated. The intervention results of these measurements have been reported previously (Bergman et al., 2018).

### Statistical Methods

#### Effects of the Intervention

We used an intention-to-treat approach for our analyses. Mixed models with maximum likelihood estimation were used to investigate group by time interactions on cognitive function, hippocampal volume, and PFC thickness. Variance components were used as the covariance structure. To report changes between the groups at each time point and within the groups at each time point compared to baseline, estimated means and pairwise comparisons of marginal means were calculated. The model included group (intervention, control), time point (baseline, 6 months and 13 months for cognitive tests; baseline and 13 months for MRI), and sex (woman, man) as fixed effects. Age at baseline was used as a covariate and participant as a random intercept for all models. For the models on hippocampal volume and PFC thickness, intracranial volume was also included as a covariate. Two-way interactions between group and time point were analyzed. In all tests, the significance level alpha was set to 0.05. We performed a total of five models for the cognitive tests and MRI, respectively; therefore, we considered a level of *p* < 0.01 as a significant effect (0.05/5). If significant changes were found in any of the MRI readouts, correlation analyses (using Spearman’s Rho) were performed with changes in cognitive performance between baseline and 13 months. Separate mixed model analyses of the right and left hemispheres are reported in Supplementary Table S1. We performed a total of eight models for the analyses of the left and right hemispheres; thus, we considered a level of *p* < 0.006 as significant for these analyses (0.05/8). All mixed model analyses were performed using SPSS version 24 (IBM statistics).

#### Longitudinal Mediation Analyses

Mediation analyses were performed on the entire MRI sample between changes in mean daily walking time (measured with the activPAL), hippocampal volume, and BDNF levels between baseline and 13 months using the lavaan package in R (Rosseel, 2012). In the models, we tested whether BDNF mediated a positive effect of daily walking time on hippocampal volume (indirect effect) using a set of linear regressions and maximum likelihood estimation. Similarly, we further tested whether the intensity level of the physical activity had different mediating effects, and thus, mediation analyses were performed between changes in mean daily time in LPA and MVPA (measured with the ActiGraph), hippocampal volume and BDNF between baseline and 13 months.

#### Cross-sectional Analyses

For exploratory analyses of the baseline data, OPLS models were calculated using percent sitting time as the response to finding associations to the body and metabolic measures, activPAL- and ActiGraph measures, cognitive function as well as measures of hippocampal volume and PFC thickness. Data inspection was carried out using PCA before OPLS analyses to detect outliers, trends, and groupings that might interfere with result interpretation. A complete list of variables included in the OPLS-models is presented in the Supplementary Material. Hippocampal volume and PFC thickness measures were adjusted for intracranial volume before these analyses, by regression on intracranial volume in each brain region. Since the effects of exercise on brain health primarily have been confirmed in older individuals (Erickson et al., 2011; Jonasson et al., 2017), age-subgroup analyses were performed, dividing participants into two groups using median split by age. OPLS-models were then created for each age group. All models were validated based on analysis of variance (ANOVA) of the cross-validated OPLS scores (CV-ANOVA) for significance testing (Eriksson et al., 2008). All OPLS-analyses were performed using SIMCA v.15.02.

## Results

Table 1 shows demographic data for the participants at baseline. Demographic data for the whole sample has been reported previously (Bergman et al., 2018). A total of six participants from the intervention group and three participants from the control group dropped out of the study during the 13 months study period. More information about missing data and dropouts is given in the Supplementary Material.

**Table 1:** Demographic data at baseline for the whole study sample and the MRI sample.

	Whole sample		MRI sample	
	Intervention (*n* = 40)	Control (*n* = 40)	Intervention (*n* = 19)	Control (*n* = 21)
Age (years), mean (SD)	52.4 (6.8)	50.3 (6.7)	51.8 (7.0)	47.0 (6.3)
Males/Females, *n* (%)	18/22 (45/55)	18/22 (45/55)	10/9 (53/47)	8/13 (38/62)
BMI (kg/m^2^), mean (SD)	29.3 (3.8)	28.9 (2.5)	29.6 (3.8)	29.0 (2.3)
Number of children, mean (SD)	2 (0.9)	2 (0.9)	2.0 (0.8)	2.1 (1.0)
Missing, *n*	0	1	0	0
Education level, *n* (%)				
Compulsory	0 (0)	2 (5)	0 (0)	1 (4.8)
Upper secondary	15 (37.5)	15 (37.5)	6 (31.6)	6 (28.6)
University	18 (45)	18 (45)	9 (47.4)	12 (57.1)
Other tertiary	7 (17.5)	4 (10)	4 (21.1)	2 (9.5)
Missing	0 (0)	1 (2.5)	0 (0)	0 (0)
Marital status, *n* (%)				
Married/living together	38 (95)	34 (85)	19 (100)	20 (95.2)
Missing	0 (0)	1 (2.5)	0 (0)	0 (0)
Self-reported health, *n* (%)				
Excellent	1 (2.5)	1 (2.5)	0 (0)	0 (0)
Very good	16 (40)	15 (37.5)	11 (57.9)	7 (33.3)
Good	14 (35)	20 (50)	5 (26.3)	13 (61.9)
Fair	6 (15)	3 (7.5)	2 (10.5)	1 (4.8)
Bad	3 (7.5)	0 (0)	1 (5.3)	0 (0)
Missing	0 (0)	1 (2.5)	0 (0)	0 (0)
Physical exercise, *n* (%)				
Never	8 (20)	6 (15)	3 (15.8)	2 (9.5)
Not regularly	8 (20)	9 (22.5)	4 (21.1)	4 (19.0)
1 time/week	6 (15)	6 (15)	2 (10.5)	5 (23.8)
2–3 times/week	14 (35)	10 (25)	7 (36.8)	5 (23.8)
>3 times/week	4 (10)	8 (20)	3 (15.8)	5 (23.8)
Missing	0 (0)	1 (2.5)	0 (0)	0 (0)

### Effects of the Intervention

The intervention was effective at increasing time spent walking, as measured with the activPAL. As previously reported when analyzing the entire study sample, time spent walking increased by 18 min (95% CI 9–26 min) at weekdays in the intervention group between baseline and 13 months compared to a 1 min (95% CI −7 to 9 min) increase in the control group, with between-group differences of 22 min (95% CI 7–37 min) at 13 months (Bergman et al., 2018). Similarly using mixed models with the group, time and day of week interactions, we further observed an interaction effect for time spent walking (*p* = 0.003), when analyzing the sub-sample randomized to the MRI measurements, with a between-group difference of 22 min (95% CI 2–42 min) at weekdays at 13 months (*p* = 0.028).

**Figure 1:**
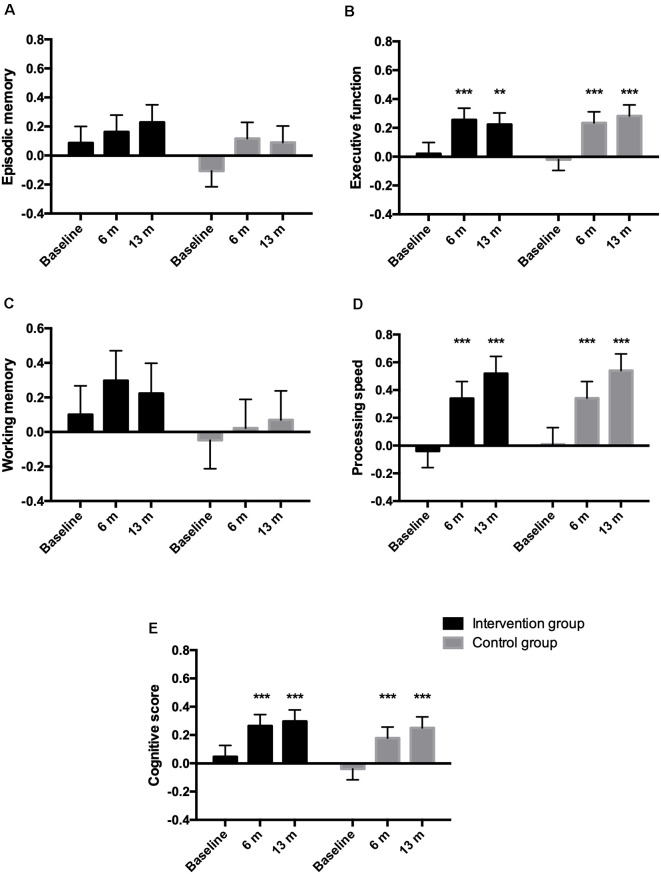
Estimated means (SEM) of z-standardized scores for the different cognitive domains episodic memory **(A)**, executive function **(B)**, working memory **(C)**, processing speed **(D)**, and for the composite cognitive score **(E)**. The cognitive score was derived from combining the different cognitive domains. **p < 0.01, ***p < 0.001 within-group difference from baseline.

No group by time interactions were observed for the different cognitive domains or global cognitive function (Figure 1). Both groups increased their performance equally regarding executive function and processing speed at the 13-month follow-up compared to baseline. For episodic and working memory, no differences were observed within any of the groups compared to baseline. Supplementary Table S2 shows the number of participants included and the mean *z* score values for the cognitive domains and the global cognitive score.

The number of participants included in the different MRI measurements is reported in Table 2. No group by time interactions were observed for hippocampal volume, vLPFC, or ACC thickness or circulating BDNF levels (Table 2). A minor, albeit significant, decrease in dLPFC thickness was observed, with a decreased thickness of 0.03 mm observed at 13 months compared to baseline within the intervention group, resulting in a significant group by time interaction (*p* = 0.01, *η*^2^ = 0.03). No difference in dLPFC thickness was observed between the groups before or after the intervention. One individual within the intervention group showed a reduced dLPFC thickness with more than 2.5 standard deviations (Supplementary Figure S1). When removing this person from the analysis the observed effect was no longer significant (*p* = 0.02). No correlations were observed between the changes in dLPFC and changes in cognitive functions between baseline and 13 months. Notably, although not significant after correction for multiple testing, there appears to be a rather pronounced difference between the left and right ACC (Supplementary Table S1).

**Table 2:** Effects of the intervention.

Outcome	Difference within groups					
	Intervention	*n*	Control	*n*	Difference between groups	Group by time interaction effects
Hippocampus (mm^3^) Baseline Difference at 13 m	8,496 (8,120, 8,871) −50 (−119, 19)	18 16	8,771 (8,422, 9,120) −17 (−82, 48)	21 18	−275 (−808, 258) −308 (−842, 226)	*F*_(1, 33.943)_ = 0.490 *p* = 0.489
dLPFC (mm) Baseline Difference at 13 m	2.74 (2.69, 2.79) −0.03 (−0.05, −0.01)**	18 16	2.72 (2.67, 2.76) 0.003 (−0.02, 0.02)	21 18	0.02 (−0.05, 0.1) −0.01 (−0.09, 0.06)	*F*_(1, 34.594)_ = 7.424 *p* = 0.010
vLPFC (mm) Baseline Difference at 13 m	2.68 (2.64, 2.72) −0.03 (−0.05, −0.01)**	18 16	2.67 (2.64, 2.71) −0.01 (−0.03, 0.01)	21 18		*F*_(1, 34.761)_ = 1.923 *p* = 0.174
ACC (mm) Baseline Difference at 13 m	2.66 (2.61, 2.72) −0.02 (−0.04, −0.001)*	18 16	2.67 (2.62, 2.72) 0.001 (−0.02, 0.02)	21 18	−0.01 (−0.08, 0.07) −0.03 (−0.1, 0.1)	*F*_(1, 34.240)_ = 2.667 *p* = 0.112
BDNF (ng/ml) Baseline Difference at 6 m Difference at 13 m	36.5 (34.1, 38.9) 0.5 (−0.7, 1.7) 0.01 (−1.2, 1.2)	39 34 33	36.8 (34.4, 39.2) −0.9 (−2.1, 0.2) −0.6 (−1.8, 0.6)	40 38 37	−0.3 (−3.7, 3.1) 1.1 (−2.3, 4.6) 0.3 (−3.2, 3.8)	*F*_(2, 143.011)_ = 1.372 *p* = 0.257

Within-group differences at each follow-up compared to baseline and between-group differences at each follow-up presented as estimated means with 95% confidence intervals, and overall test for interaction with F (degrees of freedom)—and p-values. dLPFC, dorsolateral prefrontal cortex; vLPFC, ventrolateral prefrontal cortex; ACC, anterior cingulate cortex; BDNF, brain-derived neurotrophic factor. *p < 0.05 within-group difference from baseline value. **p < 0.01 within-group difference from baseline.

### Longitudinal Mediation Analysis

**Figure 2:**
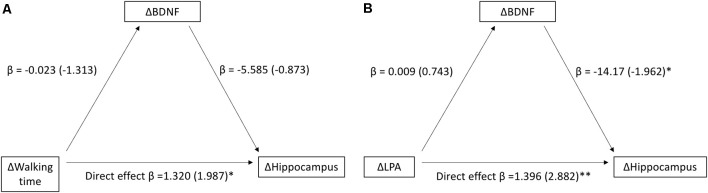
Relationship between changes in walking time and Hippocampus **(A)** and changes in light-intensity physical activity (LPA) and Hippocampus **(B)** between baseline and 13 months, as mediated by changes in BDNF between baseline and 13 months. Beta-values along with standardized coefficients (z-values within parentheses) are reported, *p < 0.05, **p < 0.01.

The mediation analysis for the entire MRI sample revealed a positive relationship between changes in walking time and changes in hippocampal volume (*n* = 34) between baseline and 13 months (Total model *β* = 1.448, *z* = 2.208, *p* = 0.027). Changes in BDNF were not related to changes in hippocampal volume, and there was no mediation effect of BDNF in this model (*β* = 0.127, *z* = 0.727, *p* = 0.467; Figure 2A). When further investigating putative mediating effects of the different intensity levels (LPA, MVPA), a positive relationship between changes in LPA and changes in hippocampal volume (*n* = 33, Total model *β* = 1.274, *z* = 2.510, *p* = 0.012) was observed. Changes in BDNF levels were weakly related to changes in hippocampal volume and did not mediate an effect of LPA on hippocampal volume *β* = −0.122, *z* = −0.695, *p* = 0.487; Figure 2B). We did not observe any relationships between MVPA, BDNF, and hippocampal volume. Participants were further assigned to younger and older age groups using the same median split value as in the OPLS analyses described below, i.e., 51 years. In these analyses, no significant relationship was found between changes in walking time and changes in hippocampal volume in either age-group (Supplementary Figure S2). The positive relationship observed between changes in LPA and changes in hippocampal volume between baseline and 13 months was driven by the older half of the sample (Supplementary Figure S3). A scatter plot depicting the changes in hippocampal volume and walking time in each group is presented in the Supplementary Figure S4.

### Cross-sectional Analysis

**Figure 3:**
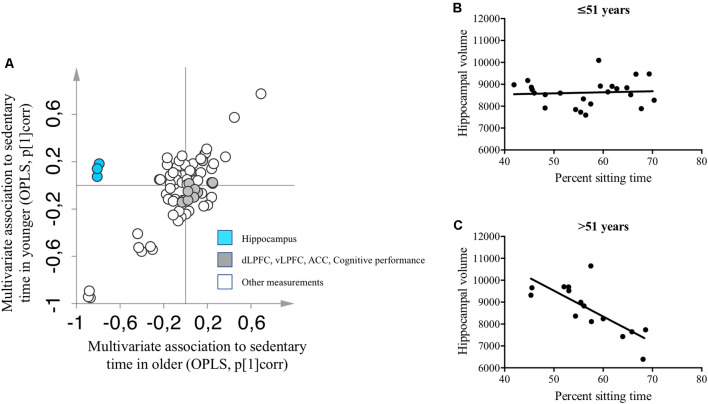
**(A)** Two separate OPLS models describing baseline associations between measures of brain and cognitive functions and percentage sitting time in participants younger (y-axis) and older than 51 years (x-axis). A complete list of variables included in the OPLS-models is presented in the Supplementary Material. **(B)** Univariate linear regression analysis between hippocampal volume and percentage sitting time among participants younger than 51 years. **(C)** Univariate linear regression analysis between hippocampal volume and percentage sitting time among participants older than 51 years. OPLS, orthogonal partial least squares; pcorr = correlation scaled OPLS weights, i.e., multivariate association to percent sitting time. ACC, anterior cingulate cortex; dLPFC, dorsolateral prefrontal cortex; vLPFC, ventrolateral prefrontal cortex.

We observed a significant OPLS model for the whole study sample (*n* = 79; CV-ANOVA *p* < 0.001). Median split by age created two groups with participants aged ≤51 years old in one group (*n* = 40, mean age 45.6 ± 3.7 years) and participants >51 years of age in the other group (*n* = 39, mean age 57.2 ± 3.2 years). No outliers were found using PCA inspection (data not shown). We observed significant age-specific OPLS models that describe the association between the percentage of time spent sitting (the response) and variables describing brain function, body measures and composition, and metabolic status (CV-ANOVA *p* < 0.001). The percentage of time spent sitting was negatively correlated with the left, right, and total hippocampus volume among participants older than 51 years, but not among participants younger than 51 years (Figure 3A). Results obtained from the multivariate models were validated employing univariate linear regression analyses. Similar to the OPLS models, we observed a strong negative association among participants older than 51 years between percentage sitting time and hippocampal volume (adjusted *R*^2^ = 0.553, standardized *β* = −0.765, *p* < 0.001), while no association was observed among participants younger than 51 years (adjusted *R*^2^ = −0.034, standardized *β* = 0.103, *p* = 0.633; Figures 3B,C).

## Discussion

To our knowledge, this is the first RCT investigating long-term effects of increased walking time at offices on cognitive function, PFC thickness, hippocampal volume, and BDNF levels. We found no effects on cognitive function or brain structure by installing treadmill workstations in offices in this RCT, in line with results of a previous non-randomized shorter study using bike desks, where no effects on short-term memory, selective attention, response inhibition or sustained attention were observed (Torbeyns et al., 2016). Notably, changes in walking time and in LPA between baseline and 13 months were positively associated with changes in hippocampal volume. Further, our exploratory analysis of the baseline data interestingly revealed that percentage sitting time was negatively associated with hippocampal volume in participants older than 51 years.

A decreased dLPFC thickness was observed for the intervention group. The effect size of the interaction was in the small range (*η*^2^ = 0.03), and it was driven by one participant showing a decreased thickness of more than 2.5 standard deviations. When removing this outlier from the analysis, the effect was no longer significant. Further, the decrease in dLPFC thickness did not correlate to changes in any of the measurements of cognitive function, indicating that this reduced thickness may not be of any functional importance.

In interventions including aerobic or muscle-strengthening physical activity, significant albeit modest effects on cognitive functions have consistently been observed (Physical Activity Guidelines Advisory Committee, 2018). Notably, there was a decrease in MVPA levels in both groups in our study, especially within the intervention group at weekends (Bergman et al., 2018). It is thus possible that the observed decrease in MVPA reduced the potential gain on cognitive function and brain structure from increased walking time. It is also important to consider that our study population was healthy, middle-aged, and relatively active already at baseline (Bergman et al., 2018). It cannot be ruled out that increasing walking time in subjects with metabolic alterations, e.g., type 2 diabetes mellitus, may induce positive effects on cognitive function and/or brain structure. Further, we did not observe any changes in weight or other anthropometric variables during the study (Bergman et al., 2018), which may have prevented positive effects on the cognitive and brain measurements.

The mediation analyses showed that changes in walking time and LPA were positively associated with changes in hippocampal volume, although not mediated by BDNF. It has previously been reported that LPA and MVPA may have different effects on cardio-metabolic risk markers (Duvivier et al., 2018), and the effects on BDNF might also differ between these different intensity levels of physical activity, which needs to be further investigated. Notably, a 10-min-long bout of very light-intensity exercise has been associated with elevated activity in hippocampal subfields and the functional connectivity between hippocampal and cortical regions (Suwabe et al., 2018). Thus, functional connectivity changes from interventions aiming to reduce sitting time in the long term warrants further investigation.

Multivariate analysis showed that more sitting time was associated with a smaller hippocampal volume. Notably, this association was only observed within the group of participants over 51 years of age. In line with this, positive associations have previously been reported between total gray matter volume and sensor-measured physical activity, but only among adults over 60 years of age (Hamer et al., 2018). Further, while evidence of the positive long-term effects on cognition and the brain of MVPA is moderately strong among adults aged 50 years or older, the evidence is insufficient among adults younger than 50 years of age (Physical Activity Guidelines Advisory Committee, 2018). Whether efforts to reduce sitting time and increase physical activity, especially LPA, can prevent hippocampal atrophy and promote healthy aging, and whether putative effects differ between age-groups, is unknown but is certainly of interest for future studies. However, the findings from the exploratory analyses were based on cross-sectional data from baseline measurements, so causality cannot be addressed.

### Strengths and Limitations

The strengths of this study include the randomized controlled design, the long-term follow up, and the relatively large sample size for the cognitive tests. The cognitive tests that we used in this study were based on a previously used larger test battery, where a five-factor analysis with loadings from the same domains as used in our study showed a good fit and good reliability (Jonasson et al., 2017). The use of OPLS statistics allowed us to investigate our data with a multivariate approach, making it possible to examine relationships between several interrelated variables simultaneously. Furthermore, sedentary behavior and physical activity were measured using two different accelerometers, reducing the risk of e.g., recall bias apparent when using subjective measurements.

The increase in walking time observed in the intervention group was higher during the initial phase of the study period (Bergman et al., 2018). Performing the MRI measurements also at an earlier stage of the study period could thus have captured an early response in hippocampal volume. Our initial hypothesis according to our previously published study protocol was that LPA would increase in the intervention group throughout the study period compared to the control group (Bergman et al., 2015). However, the LPA pattern over the intervention period of 13 months was rather complex, with an initial 2 months increase during weekdays within the intervention group, but a reduction on weekends at the end of the study period (Bergman et al., 2018). This implies a complex change in behavior, affecting the interpretation of our brain and cognitive measurements. The participants were told to use the treadmill for at least 1 h per day, distributed as they preferred during the day. Perhaps informing the participants to break up their sitting time more often, rather than only reducing the total sitting time *per se*, would have given different results on cognitive function and brain structure, as regular breaks from sitting have been shown to improve e.g., blood glucose levels (Dempsey et al., 2017), a potential mediating mechanism between LPA and brain health (Wheeler et al., 2017). The design of the treadmill did not make it possible to track how much and at which speed the participants used it. Further, it was only possible to perform the MRI measurements on a subset of the study population; including more participants would have increased the power of these calculations and the corresponding mediation analyses. It has been suggested that cardiovascular fitness is related to brain networks relevant for healthy brain aging independent of habitual levels of physical activity, highlighting the importance of measuring both physical activity and cardiorespiratory fitness (Voss et al., 2016). However, in the present study, we could not measure cardiorespiratory fitness. As the test-retest effects are strong within short time-periods, this may contribute to the lack of effects on cognitive function observed in our study. Finally, the power calculation was based on time spent walking. It is thus possible that the study was underpowered to find any effects regarding cognitive functions and brain structure.

## Conclusions

Even though no group by time interactions could be observed on cognitive performance or brain morphology from installing treadmill workstations in offices, our results imply that increased walking time and LPA may have a positive effect on brain volume and that reduced sedentary behavior could be associated with brain structures of importance for memory functions. Strategies to increase LPA and decrease sitting time may thus have a positive impact on brain health, but this, together with mediating mechanisms, needs to be further evaluated in long-term studies.

## Data Availability Statement

The data can be shared considered on an individual basis upon reasonable request from the corresponding author.

## Ethics Statement

The studies involving human participants were reviewed and approved by Regional Ethical Review Board, Umeå, Sweden. The patients/participants provided their written informed consent to participate in this study.

## Author Contributions

FB, AS, PW, JL, TO, and C-JB planned and designed the study. FB and C-JB collected the cognitive and MRI-data. FB, LJ, and MR processed the MRI-data. FB, TM-F, and MR analyzed cognitive and MRI data. LJ performed the mediation analyses. TM-F and EC performed the multivariate analyses. FB processed the accelerometer data with assistance from PW, FÖ, and TO. FB wrote the first draft of the manuscript and the revised versions. All authors contributed to the article and approved the submitted version.

## Conflict of Interest

The authors declare that the research was conducted in the absence of any commercial or financial relationships that could be construed as a potential conflict of interest.
