# Supplementary material for: Walking Time Is Associated With Hippocampal Volume in Overweight and Obese Office Workers
Source: Front Hum Neurosci. 2020 Aug 20;14:307. doi: 10.3389/fnhum.2020.00307 (PMC7468418; doi:10.3389/fnhum.2020.00307)
Supplement: Supplementary file 1 [file Data_Sheet_1.pdf]

Supplementary material

The cognitive test battery

Episodic memory

Free recall

In this test, a list of 16 Swedish nouns was shown on a computer screen for three seconds per word. The task was, immediately after the test, to write down as many of the words shown as possible on a blank sheet during a maximum of two minutes, with the number of correctly recalled words as the dependent variable

Word recognition (encoding – retrieval)

During the first phase (the encoding phase), a list of 30 Swedish nouns was shown for three seconds each on a computer screen. The task was to encode the words for the later retrieval phase, which took place about 25 minutes after the encoding phase. During the retrieval phase, the participants were given a new list of words, consisting of 15 words from the encoding phase and 15 new words. The task was, within three seconds, to indicate whether the word currently shown had been shown during the encoding phase or not. Participants received one training session consisting of five words, before starting the real trial. The dependent variable was the percentage of correctly recalled words.

Executive function

N-back (1-back, 2-back, 3-back)

In these three separate tests, participants were told to, within two seconds from stimuli onset, indicate by a key press on the computer whether the number currently shown on the computer screen matched the number that was shown one, two or three numbers back, respectively. In total 20 numbers were shown per task. Participants received one training session with 1-back consisting of a session with four numbers, before starting the real trial. The dependent variable was 1-, 2- and 3-back accuracy, measured as percent correct numbers.

Flanker test

In this test, five arrows were shown on a computer screen for two seconds each. Participants were told to indicate whether the mid arrow in either a congruent (<<<<< or >>>>>) or incongruent (<<><< or >><>>) trial pointed to the left or right by pressing on the left or right arrow on the computer keyboard as quickly and accurately as possible. In 50% of the trials, the arrows were congruent, and in the other 50% of the trials, the arrows were incongruent. The participants received one training session before the real trial. The dependent variable was the time difference between congruent and incongruent trials.

Trail making 2 (TMT2) and trail making 4 (TMT4)

In the pen and paper D-KEFS trail making task series 2 and 4, participants were shown circles containing numbers (TMT2) and circles containing numbers and letters (TMT4). The task in TMT2 was to draw lines between the circles to connect the numbers 1 to 16 in numerical order as quickly as possible. In TMT4, the task was to draw lines between the circles, alternating between numbers (1 to 16) and letters (A to P), in numerical and alphabetical order, as quickly as possible (number 1 to letter A, letter A to number 2, number 2 to letter B, and so on). Training sessions, where the participants were supposed to draw lines between circles to connect the numbers 1 to 5 in numerical order (before TMT2) and alternating between numbers (1 to 4) and letters (A to D), were given before the real trial started. The

dependent variable was points given based on the time to complete the test according to the scoring manual for the two tests, respectively (Delis et al., 2001).

Working memory

Backward digit span (BWDS)

In this computerized version of the Wechler Adult Intelligence Scale-Revised (WAIS-R) digit span backward task, the participants were told to remember a line of digits between 1 and 9 in the reverse order of how they were shown. The digits were shown on the middle of the screen for one second each, and the participants gave their answers by pressing the correct number on the keyboard. Participants received two training sessions, each consisting of two sessions with two numbers, before starting the real trial. The first level of the real trial consisted of three numbers. If the response given by the participant was correct, the difficulty increased by one number until reaching the maximum of nine levels. If the response given by the participant was incorrect, the same digit sequence length was shown again. If two incorrect responses were given, the test ended. The dependent variable was the highest achieved level.

Processing speed

Similarities

In this pen-and-paper test, the participants were asked to visually examine whether any of two target symbols matched five other symbols. If there was a match, the participants were asked to draw a line over the matched symbol, if there was no match the participants were asked to draw a line over the "no" symbol. Participants received a training session before the real trial. The maximum time limit for the real trial was 120 seconds. The dependent variable for the similarities task was the number of completed items.

Digit symbol

In the pen-and-paper version of the WAIS-R Digit Symbol task, the participants were told to draw symbols in empty boxes associated with numbers (between 1–9). Each number was associated with a specific symbol according to a key that was visible throughout the task. They had 120 seconds to finish as many digit-symbol pairs as possible. For the computerized version, the participants were shown a key of all the symbols with matching numbers on a computer screen. Below the key, the participants were shown one symbol and one number, and the task was to indicate whether this combination was similar to the key above. They indicated whether there was a match by using the arrows on the keyboard (left arrow yes and right arrow no). There was either no delay or a 4-second time delay from when the key appeared on the screen and the target digit-symbol pair. The dependent variable for the pen- and paper version was the number of completed items, and for the computerized version the dependent variable was the reaction time for zero- and four-seconds delay, respectively.

References

Delis, D.C, Kaplan, E., and Kramer, J.H. 2001. *Delis-Kaplan Executive Function System*. San Antonio, TX: The Psychological Corporation.

List of included variables in the orthogonal partial least squares (OPLS)- models

Response variable

- Percent sitting time

Sedentary behavior and physical activity variables

- Mean daily standing time
- Mean daily walking time
- Mean daily steps
- Mean daily percent standing time
- Mean daily number of breaks from sitting
- Mean daily sitting duration
- Mean daily time in prolonged sitting (>30 minutes)
- Mean daily break rate (number of breaks per sedentary hour)
- Mean daily number of breaks 0 – 3 minutes long
- Mean daily number of breaks over 20 minutes long
- Mean daily total time in moderate to vigorous physical activity
- Mean daily percent in moderate to vigorous physical activity
- Mean daily total time in light-intensity physical activity
- Mean daily percent in light-intensity physical activity

Cognitive functions, hippocampal (HC) volume and prefrontal cortex (PFC) thickness

- Executive functions
- Episodic memory
- Working memory
- Processing speed
- Global cognitive score
- Left HC volume
- Right HC volume
- Total HC volume
- Left dorsolateral PFC thickness
- Right dorsolateral PFC thickness
- Mean dorsolateral PFC thickness
- Left ventrolateral PFC thickness
- Right ventrolateral PFC thickness
- Mean ventrolateral PFC thickness
- Left anterior cingulate cortex thickness
- Right anterior cingulate cortex thickness
- Mean anterior cingulate cortex thickness

Body measurements, body composition and metabolic parameters

- Height
- Weight
- BMI
- Waist circumference
- Hip circumference
- Sagittal height
- Systolic blood pressure

- Diastolic blood pressure
- Pulse
- ASAT
- ALAT
- ApoBT
- ApoAT
- Triglycerides
- Total cholesterol
- Fasting glucose
- Insulin levels from oral glucose tolerance test at 0, 30, 60, 90 and 120 minutes
- Total area under the curve for insulin
- HOMA – IR
- HbA1c
- CRP
- Total body mass
- Percent fat mass
- Total fat mass
- Total lean mass
- Percent android fat mass
- Percent gynoid fat mass
- BDNF

Salivary cortisol

- Cortisol measured at 07.00 am
- Cortisol measured at 11.00 am
- Cortisol measured at 04.00 pm
- Cortisol measured at 11.00 pm
- Area under the curve for cortisol between 07.00 – 11.00 am
- Area under the curve for cortisol between 11.00 am – 04.00 pm
- Area under the curve for cortisol between 04.00 pm – 11.00 pm
- Total area under the curve cortisol
- Cortisol slope

Other measurements

- Sex
- Educational level
- Hospital anxiety and depression scale – depression and anxiety subscales

Missing data and dropouts

One participant in the intervention group developed a medical condition during baseline and dropped out of the study, and the baseline values for this participant were therefore removed from the analyses. In the intervention group, two more participants dropped out of the study before the 6 months measurement, and three more participants dropped out of the study before the 13 months measurement. Further in the intervention group, two participants did not perform the 6 months measurement and one participant did not perform the 13 months measurement due to temporary sick-leave. In the control group, two participants dropped out of the study before the 6 months measurement and one more participant dropped out of the study before the 13 months measurement.

One subject in the intervention group had missing values for the word recognition test at baseline, and a score for episodic memory and cognitive score could thus not be calculated for this person at this time-point. In the intervention group, one participant did not do any MRI-measurement at baseline or 13 months due to claustrophobia during scanning, one participant in the intervention group did not do the MRI-measurements at 13 months due to temporary sick-leave and one participant had dropped out of the study before the 13 months measurement. In the control group, one participant could not do the MRI-measurement at 13 months due to scanner malfunctioning, one participant had dropped out of the study and one participant did not want to do the MRI-scan at 13 months. One subject in the intervention group was removed from the BDNF calculations at 6 months due to unreasonable values indicating measurement error.

Supplementary Table 1. Mixed model analyses of hippocampal volume and PFC thickness in the left and right hemisphere.

Outcome	Difference within groups				Difference between groups	Group by time interaction effects
	Intervention	n	Control	n		
**Left Hippocampus [mm^3]**						
Baseline	4180 (4003, 4357)	18	4337 (4173, 4501)	21	-157 (-407, 94)	$F_{(1, 33.972)} = 1.228$
Difference at 13 m	-17 (-61, 27)	16	16 (-25, 57)	18	-189 (-441, 62)	p = 0.276
**Right Hippocampus [mm^3]**						
Baseline	4315 (4108, 4522)	18	4434 (4242, 4626)	21	-118 (-412, 175)	$F_{(1, 33.923)} = 0.002$
Difference at 13 m	-32 (-69, 5)	16	-33 (-68, 2)	18	-117 (-411, 176)	p = 0.969
**Left dLPFC [mm]**						
Baseline	2.76 (2.70, 2.81)	18	2.72 (2.66, 2.77)	21	0.04 (-0.04, 0.1)	$F_{(1, 34.416)} = 3.330$
Difference at 13 m	-0.03 (-0.05, -0.01)**	16	-0.004 (-0.02, 0.02)	18	0.02 (-0.06, 0.09)	p = 0.077
**Right dLPFC [mm]**						
Baseline	2.72 (2.67, 2.77)	18	2.72 (2.67, 2.76)	21	0.01 (-0.07, 0.08)	$F_{(1, 34.628)} = 10.266$
Difference at 13 m	-0.04 (-0.06, -0.02)**	16	0.01 (-0.01, 0.03)	18	-0.04 (-0.1, 0.03)	p = 0.003
**Left vLPFC [mm]**						
Baseline	2.70 (2.67, 2.74)	18	2.68 (2.64, 2.71)	21	0.03 (-0.03, 0.08)	$F_{(1, 34.253)} = 0.651$
Difference at 13 m	-0.02 (-0.04, -0.01)*	16	-0.01 (-0.03, 0.01)	18	0.02 (-0.04, 0.07)	p = 0.425
**Right vLPFC [mm]**						
Baseline	2.65 (2.61, 2.70)	18	2.67 (2.62, 2.71)	21	-0.01 (-0.08, 0.05)	$F_{(1, 35.043)} = 2.675$
Difference at 13 m	-0.04 (-0.06, -0.01)**	16	-0.01 (-0.03, 0.02)	18	-0.04 (-0.11, 0.03)	p = 0.111
**Left ACC [mm]**						
Baseline	2.67 (2.62, 2.73)	18	2.68 (2.63, 2.73)	21	-0.01 (-0.09, 0.08)	$F_{(1, 34.329)} = 0.001$
Difference at 13 m	-0.003 (-0.03, 0.02)	16	-0.003 (-0.02, 0.02)	18	-0.01 (-0.09, 0.08)	p = 0.972
**Right ACC [mm]**						
Baseline	2.65 (2.60, 2.71)	18	2.66 (2.61, 2.71)	21	-0.01 (-0.09, 0.07)	$F_{(1, 34.488)} = 5.961$
Difference at 13 m	-0.03 (-0.06, -0.01)**	16	0.004 (-0.02, 0.03)	18	-0.04 (-0.1, 0.04)	p = 0.020

Number of participants at each time-point, within-group differences at each follow-up compared to baseline, between-group differences at each follow-up presented as estimated means with 95% confidence intervals, and overall test for interaction with F (degrees of freedom) - and p-values. dLPFC = dorsolateral prefrontal cortex. vLPFC = ventrolateral prefrontal cortex. ACC = anterior cingulate cortex. BDNF = brain-derived neurotrophic factor. * p<0.05 within-group difference from baseline value. ** p<0.01 within-group difference from baseline.

Supplementary Table 2. Number of participants included and mean z-scores for cognitive domains and cognitive score.

Outcome	Intervention group Mean ± SD	Number of subjects	Control group Mean ± SD	Number of subjects
Episodic memory				
Baseline	0.0916 ± 0.62	38	-0.0585 ± 0.77	40
6 m	0.1660 ± 0.75	35	0.1541 ± 0.91	38
13 m	0.2245 ± 0.65	33	0.1065 ± 0.91	37
Executive function				
Baseline	-0.0121 ± 0.55	39	0.0118 ± 0.64	40
6 m	0.2541 ± 0.34	35	0.2733 ± 0.53	38
13 m	0.2351 ± 0.52	33	0.3137 ± 0.44	37
Working memory				
Baseline	0.0243 ± 0.92	39	-0.0237 ± 1.08	40
6 m	0.2256 ± 1.07	35	0.0409 ± 1.20	38
13 m	0.1976 ± 1.18	33	0.0409 ± 1.26	37
Processing speed				
Baseline	-0.0726 ± 0.83	39	0.0707 ± 0.84	40
6 m	0.3003 ± 0.73	35	0.3884 ± 0.94	38
13 m	0.5290 ± 0.73	33	0.5896 ± 0.81	37
Cognitive score				
Baseline	0.0260 ± 0.46	38	0.0001 ± 0.59	40
6 m	0.2365 ± 0.46	35	0.2142 ± 0.67	38
13 m	0.2965 ± 0.49	33	0.2627 ± 0.52	37

Mean z-scores with SD for each group at each follow-up.

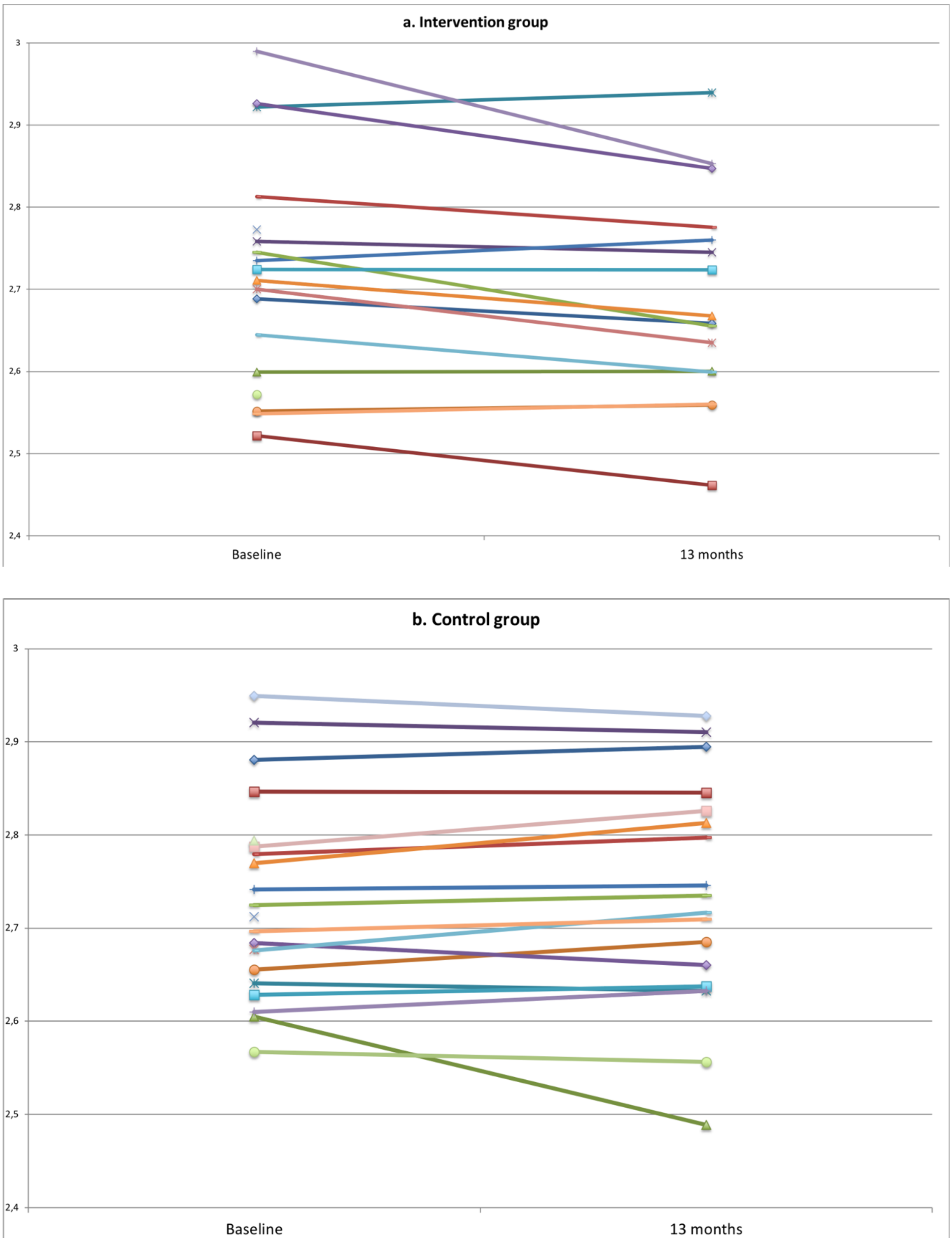

Supplementary Figure 1. Thickness of the dorsolateral prefrontal cortex (dLPFC) at baseline and 13 months for participants in the intervention group (a) and control group (b). Each line represents one person. In the z scores for the intraindividual changes between baseline and 13 months based on the entire MRI study population, one subject in Figure 1a had a decline in dLPFC cortical thickness of more than 2.5 standard deviations.

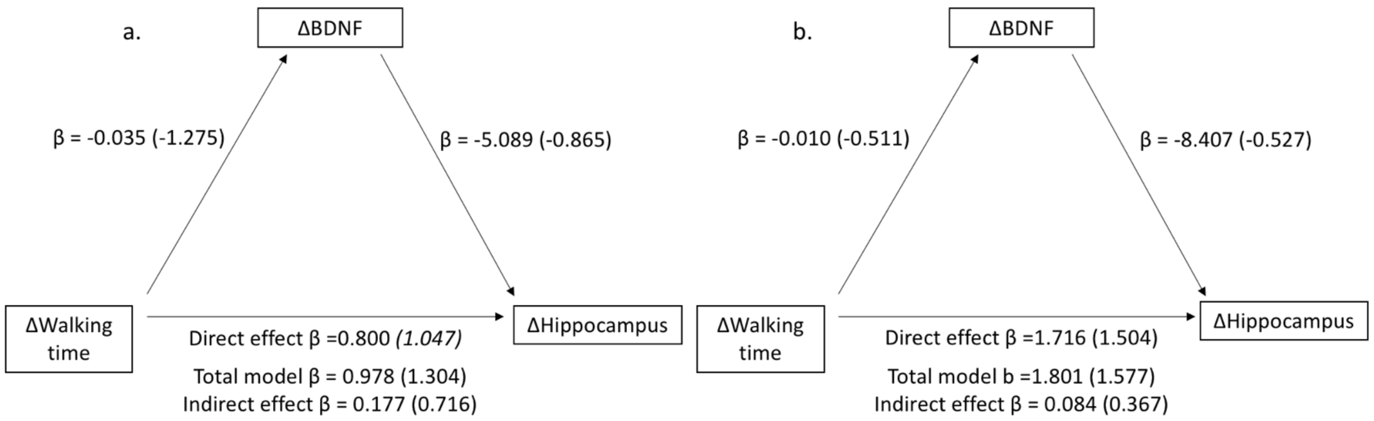

Supplementary Figure 2. Relationship between changes in walking time and Hippocampus between baseline and 13 months for (a) participants younger (n=21) and (b) older (n = 13) than 51 years of age, as mediated by changes in BDNF between baseline and 13 months. Beta-values are reported along with standardized coefficients (z-values within parentheses).

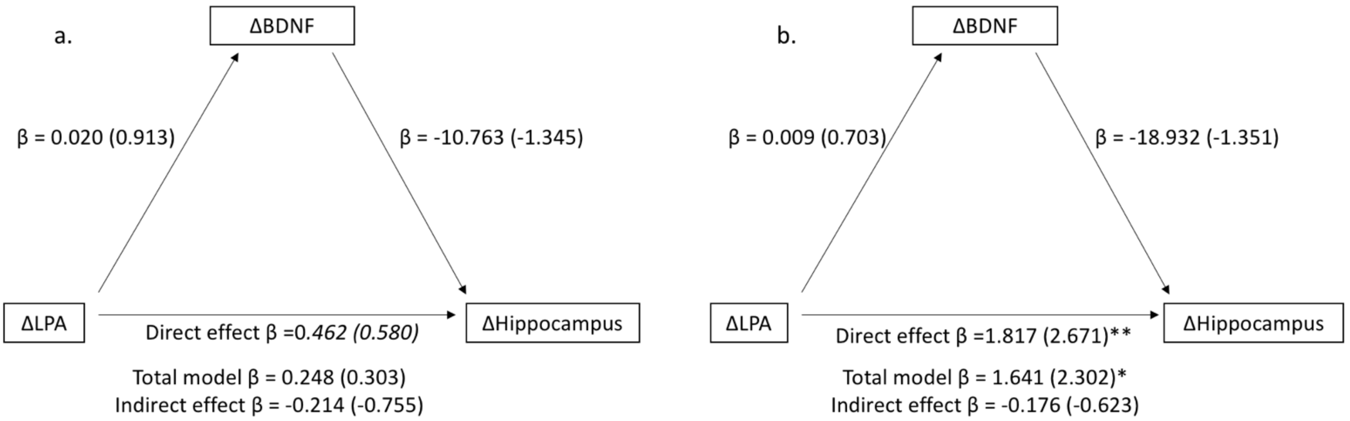

Supplementary Figure 3. Relationship between changes in light-intensity physical activity (LPA) and Hippocampus between baseline and 13 months for (a) participants younger (n=20) and (b) older (n = 13) than 51 years of age, as mediated by changes in BDNF between baseline and 13 months. Beta-values are reported along with standardized coefficients (z-values within parentheses). *p<0.05 **p<0.01.

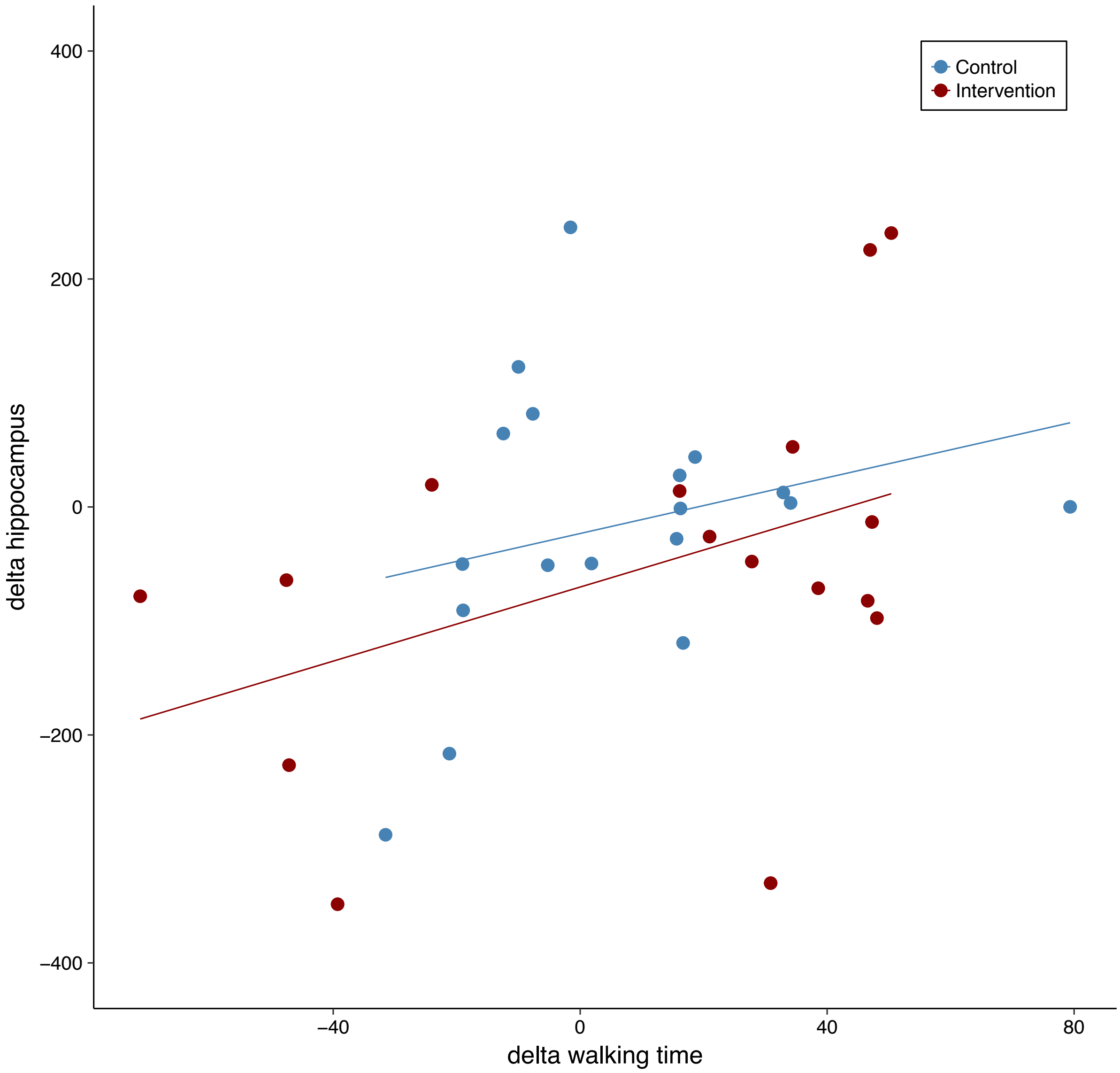

Supplementary Figure 4. Changes between baseline and 13 months in hippocampus and walking time in the intervention and control group.
